# The FunFOLD2 server for the prediction of protein–ligand interactions

**DOI:** 10.1093/nar/gkt498

**Published:** 2013-06-11

**Authors:** Daniel B. Roche, Maria T. Buenavista, Liam J. McGuffin

**Affiliations:** ^1^Laboratoire de génomique et biochimie du métabolisme, Genoscope, Institut de Génomique, Commissariat à l'Energie Atomique et aux Energies Alternatives, Evry, Essonne 91057, France, ^2^UMR 8030 – Génomique Métabolique, Centre National de la Recherche Scientifique, Evry, Essonne 91057, France, ^3^Départment de Biologie, Université d’Evry-Val-d’Essonne, Evry, Essonne 91000, France, ^4^PRES UniverSud Paris, Saint-Aubin, Essonne 91190, France, ^5^School of Biological Sciences, University of Reading, Reading, Berkshire RG6 6AS, UK, ^6^BioComputing Section, Medical Research Council Harwell, Harwell Oxford, Oxfordshire OX11 0RD, UK and ^7^Beamline B23, Diamond Light Source, Didcot, Oxfordshire OX11 0QX, UK

## Abstract

The FunFOLD2 server is a new independent server that integrates our novel protein–ligand binding site and quality assessment protocols for the prediction of protein function (FN) from sequence via structure. Our guiding principles were, first, to provide a simple unified resource to make our function prediction software easily accessible to all via a simple web interface and, second, to produce integrated output for predictions that can be easily interpreted. The server provides a clean web interface so that results can be viewed on a single page and interpreted by non-experts at a glance. The output for the prediction is an image of the top predicted tertiary structure annotated to indicate putative ligand-binding site residues. The results page also includes a list of the most likely binding site residues and the types of predicted ligands and their frequencies in similar structures. The protein–ligand interactions can also be interactively visualized in 3D using the Jmol plug-in. The raw machine readable data are provided for developers, which comply with the Critical Assessment of Techniques for Protein Structure Prediction data standards for FN predictions. The FunFOLD2 webserver is freely available to all at the following web site: http://www.reading.ac.uk/bioinf/FunFOLD/FunFOLD_form_2_0.html.

## INTRODUCTION

Proteins have an essential cellular role in all living organisms; thus, they are crucial in the maintenance of cellular and organism homeostasis. The ubiquitous role of proteins in cellular systems, make the determination of protein function (FN), ligand binding site residues and potential binding partners, essential to gain a more in-depth knowledge of cellular functionality (1–3). The predicted structure of proteins can aid in the determination of a proteins cellular function, and hence bioinformatics tools such as the FunFOLD2 server have been developed to predict protein-ligand binding via the use of 3D models (1,2).

The FunFOLD2 server integrates our cutting edge function prediction algorithms, to predict protein–ligand binding sites from a single sequence via the production of 3D structures using the IntFOLD2-TS protocol (4). The server is intended for use by both expert and non-expert users alike. Non-expert users can use the ligand-binding site predictions as a guide to the likely binding sites and potential ligands, whereas expert users can look more closely into the data provided. For submission of a query sequence, an easy-to-use web interface is available, which allows the non-expert user to predict a variety of protein function prediction features, including ligand-binding site residues for the top predicted binding site, putative binding site ligands, 3D models of the likely protein–ligand interactions, protein–ligand binding site feature scores (1) used to predict the overall quality of the prediction [predicted Matthews Correlation Coefficient (MCC) (5) and Binding-site Distance Test (BDT) (6) scores] and the probability that each proposed binding site residue binds to particular ligand types (ions, organic ligands, peptides and nucleotides). In addition, users have the option of downloading the 3D structure, which includes the superposed predicted ligands within the predicted binding site. A comprehensive help page is included for the server, containing details on the required input and output from the server and an example results page.

The original FunFOLD server (2) has been operational since late January 2011, and the outputs have been extensively used by researchers from within the UK, France [external groups at Reading (7,8) and Genoscope] and international groups during the CASP10 prediction season, which ran from April to August 2012. This article describes a novel server implementation of the FunFOLD (2) protocol that now includes improved model ranking using local binding site residue quality scores [FunFOLDQA (1)], along with the addition of new output scores. The FunFOLD2 predictions were independently validated by the CASP10 assessors using numerous performance benchmarks. The server is also being continuously evaluated as part of CAMEO (9) (Continuous Automated Model EvaluatiOn), in the ligand binding prediction category (http://www.cameo3d.org). Although other freely available servers exist for the prediction of function and ligand-binding site residues (10–17), to our knowledge, the FunFOLD2 server is the first server to directly integrate a ligand-binding site quality assessment method for use in protein function prediction.

## IMPLEMENTATION

**Figure 1.**
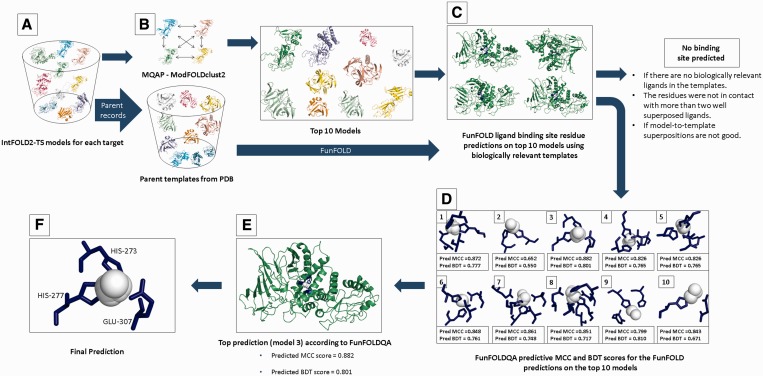
Flow diagram of the FunFOLD2 prediction server pipeline. (**A**) A number of alternative models are built for the target sequence using the IntFOLD2-TS protocol (4). (**B**) The FunFOLD2 pipeline then uses ModFOLDclust2 (18) to determine the top models for each target. (**C**) The FunFOLD algorithm (2) is subsequently used to predict ligand-binding site residues for the top models. (**D**) The quality is assessed for the resultant FunFOLD predictions, using our ligand-binding site quality assessment tool, FunFOLDQA (1). (**E**) The predicted MCC and BDT scores [according to FunFOLDQA (1)] are provided, along with the propensity of which ligand type the binding site is most likely to contain, along with ligand functional propensity. (**F**) Final prediction.

Figure 1 shows the implementation of the FunFOLD2 server, which emphasizes the interdependency between the FunFOLD (2) and the FunFOLDQA (1) algorithms. The first key step is the generation of IntFOLD2-TS models (4), ranked according ModFOLDclust2 (18) global model quality scores. The ranked models and a list of non-redundant parent templates are subsequently processed by the FunFOLD algorithm. For each model, the FunFOLD algorithm produces a list of residues from the target sequences that are most likely to bind a ligand [in Critical Assessment of Techniques for Protein Structure Prediction (CASP) FN format], along with a list of putative binding ligands. The FunFOLD results for each model, along with the model used and parent template list, are then fed into the FunFOLDQA algorithm, which assesses both global and per-residue ligand-binding site prediction quality. The FunFOLDQA algorithm outputs the predicted BDT (6) and predicted MCC (5) scores (and the component feature scores from the neural network inputs). In addition, the propensity that each predicted ligand-binding site residue is in contact with the four different ligand types is assessed [Ions (I), Organic ligands (O), Nucleotides (N) or Peptides (P)], as specified in the CAMEO (9) Ligand Binding (LB) category. Finally, predicted MCC and BDT scores are used to rank the FunFOLD predictions, outputting the top-ranked prediction as the best prediction to the web server. The use of FunFOLDQA to rank FunFOLD predictions, on the top 10 models, has been shown to result in significantly improved predictions [see Roche *et al.* (1)]. A brief overview of both the FunFOLD and FunFOLDQA algorithms are described later in the text. The new global and per-residue functional propensity metrics are described in the supplementary methods.

## FunFOLD

The FunFOLD method (2) is based on one key concept: proteins containing ligands within the PDB, with a similar fold as the 3D model of the target protein being studied, are likely to having similar binding sites (2). A ligand is defined as a biologically relevant molecule, which binds to a structurally elucidated enzyme in the PDB. The FunFOLD algorithm uses the TM-align (19) method to superpose templates containing biologically relevant ligands with the predicted 3D structure from IntFOLD2-TS. Each model-to-template superposition is subsequently used if the TM-score ≥ 0.4 [TM-scores from 0.4 to 0.6 have previously been shown to mark the transition from unrelated to significantly related folds (20)]. The superpositions are then combined and reoriented using a PyMOL script (http://www.pymol.org), to determine putative ligand clusters. An agglomerative hierarchical clustering algorithm is subsequently used to identify each continuous mass of contacting ligands, thus suggesting potential ligand binding pockets. The criteria for determining contacts between ligands are defined as less than or equal to the Van der Walls radius of an atom plus 0.5 Å. The cluster with the largest number of ligands is then selected as the location of the ligand-binding site pocket. To determine the ligand binding site residues in the selected binding pocket, a novel ‘residue voting’ algorithm is used. Residues are determined to be in contact with the ligand cluster, if the residue has at least one contact with ≥2 ligands and at least 25% of the ligands within the cluster. The criterion for determining if a residue is in contact with a ligand is a distance of less than or equal to the Van der Walls radius of an atom plus 0.5 Å. The output from the FunFOLD algorithm is a list of putative ligand-binding site residues plus a list of the ligands within the binding site cluster (2).

The FunFOLD algorithm has been extensively benchmarked on both the CASP8 and the CASP9 data sets (2). An early implementation of the algorithm was used in the CASP9 competition (2010), where it ranked amongst the top 10 methods (21). For a more in-depth description of the FunFOLD algorithm and methods benchmarking, see Roche *et al.* (2). Furthermore, the FunFOLD algorithm has recently been used in two large-scale genomic studies (7,22).

## FunFOLDQA

The FunFOLDQA algorithm (1) was developed to help determine the reliability of our FunFOLD predictions (2) by the assignment of quality assessment scores. The final binding site quality scores that the method produces are based on the MCC (5) and BDT (6) metrics, which are used for the assessment of ligand-binding site residue predictions compared with crystal structures. The FunFOLDQA method combines four binding site-dependent protein feature scores and one structural dependent feature score, using a neural network, trained on either the MCC or BDT metrics, to produce local ligand-binding site quality predictions. The five feature scores are called: ‘BDTalign, Identity, Rescaled BLOSUM62, Equivalent Residue Ligand Distance and Model Quality’. The ‘BDTalign score’ establishes the distance between residues that are equivalent between the model binding site and each template binding site. The Identity score compares binding site residues between the model- and template-binding site, which are ‘equivalent’ in 3D space, according to their amino acid sequence. The ‘Rescaled BLOSUM62’ score is similar to the ‘Identity’ score, but it scores equivalent residues between model and template binding site, using the BLOSUM62 scoring matrix. The ‘Equivalent Residue Ligand Distance score’ scores the equivalent residues between the model and the template in relation to their distance from the bound ligand. The ‘Model Quality Score’ is the global quality score for the starting model, calculated using ModFOLDclust2 (18). For a detailed description of the scoring metrics and their associated algorithms, see Roche *et al.* (1).

In addition to combining the strengths of the FunFOLD and FunFOLDQA algorithms, the FunFOLD2 server also integrates a new metric for both the global and per-residue scoring of functional propensity (See Supplementary Methods). The function is determined as the propensity of binding to specific ligands: Ions (I), Organic ligands (O), Nucleotides (N) or Peptides (P), in accordance with CAMEO LB category requirements. All quality scores are between zero and one, with scores close to one signifying a high confidence prediction and scores close to zero signifying a low confidence prediction.

## INPUTS AND OUTPUTS

The FunFOLD2 server provides an easy to use web interface for submission of jobs: the only input required is a protein sequence in single letter amino acid code. However, optionally users may provide a name for the protein sequence and an email address. On submission of the sequence to the server, a unique URL is generated for the output, which can be bookmarked. Additionally, if a user has provided an email address, an email will be sent containing a reminder of the results URL, once the job has been completed. The time for job completion is in line with similar ligand-binding site prediction servers, which can take >24 h to return results, although typically users should expect to receive their results within the same day. Several factors influence server response time including, the server load, the size of the protein sequence under analysis and the number of templates available.

**Figure 2.**
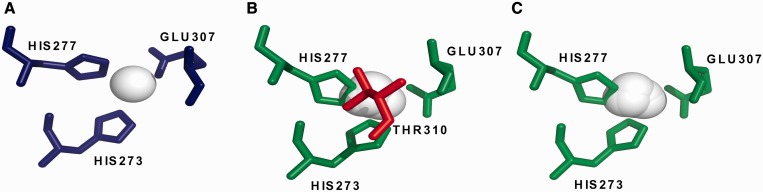
Integrating both FunFOLD (2) and FunFOLDQA (1) into the FunFOLD2 server improves predictive quality. Example of a binding site prediction from CASP10 target T0726 comparing the FunFOLD2 server with the original FunFOLD method. The green sticks represent residues in the model that have been correctly predicted as binding to the ligands. The red sticks represent residues that were incorrectly predicted as potential ligand-binding residues. The blue sticks represent the observed ligand-binding site residues in the experimental structure. The white spheres represent ligands either predicted (**B** and **C**) or observed (**A**). (A) An example of the observed CASP10 target T0726 (4fgm), with the observed binding site residues (273, 277 and 307) and ligand (ZN) shown. (B) The predicted binding site from the original FunFOLD method for T0726 with the predicted binding site residues (273, 277, 307 and 310) and ligands (ZN-8) shown, with a predicted MCC score of 0.872 and a predicted BDT score of 0.777. (C) An example where FunFOLD2 produces a perfect prediction for CASP10 target T0726 (4fgm), with the predicted binding site residues (273, 277 and 307) and ligands (ZN-8) shown. In this case, the predicted MCC score is 0.882, and the predicted BDT score is 0.801.

The server results page contains a graphical representation of the ligand-binding site, with predicted ligands and binding site residues highlighted (Figure 2), which have been rendered using PyMOL (http://www.pymol.org). Additionally, a list of ligand-binding site residues, predicted ligands and binding site residue propensities is provided in CAMEO format. Furthermore, an interactive model with predicted binding site residues and ligands can be visualized using the Jmol plug-in (http://jmol.sourceforge.net/). A link to download a PDB file of the top model with the putative ligands is also provided. If the user provides an email address on submission of their sequence, then machine-readable text files are attached to the results email in CASP-FN format and CAMEO-LB format.

## CASE STUDY—AMINOPEPTIDASE N FAMILY PROTEIN (PDB ID 4fgm AND CASP10 T0726)

The aminopeptidase N family protein Q5QTY1 from *Idiomarina loihiensis* (PDBID 4fgm and CASP10 target T0726) provides an example of output from the FunFOLD2 server (Figure 2), where using FunFOLDQA to rank predictions results in improved prediction quality. Figure 2 highlights the predicted binding site residues in green (B and C), over-predictions are shown in red and observed residues in blue (A). The ligands predicted (B and C) and observed (A) are shown in white. The top FunFOLD2 prediction correctly predicts the binding site residues (273, 277 and 307), with a predicted MCC score of 0.882 and predicted BDT score of 0.801 and per residue scores (r = histidine (HIS); n = 277; | I = 0.212; O = 000; N = 000; P = 000, r = glutamic acid (GLU); n = 307; | I = 0.335; O = 000; N = 000; P = 000, r = HIS; n = 273; | I = 0.189; O = 000; N = 000; P = 000). The top ranked prediction for the original FunFOLD method produces an over-prediction of one binding site residue (310), with a lower predicted MCC score of 0.872 and a predicted BDT score of 0.777 and pre-residue scores (r = HIS; n = 277; | I = 0.217; O = 000; N = 000; P = 000, r = GLU; n = 307; | I = 0.328; O = 000; N = 000; P = 000, r = HIS; n = 273; | I = 0.192; O = 000; N = 000; P = 000, r = threonine (THR); n = 310; | I = 0.246; O = 000; N = 000; P = 000). Furthermore, additional case studies can be found in the original benchmarking papers (1,2) and in two recently completed genomic-scale studies (7,22).

## LIMITATIONS

Predicting ligand-binding site data is a difficult task, and there are several limitations to current prediction methods. The following is a list of the most common limitations specific to the current implementation of the FunFOLD2 server: (i) If the server is unable to build a starting model for the target sequence, then it cannot predict any ligand-binding sites, although, fortunately, for the majority of protein targets, a reasonable 3D model can be obtained. (ii) If no structural similarity can be found, between the target model and structurally elucidated proteins with bound biologically relevant ligands, then a prediction is not made. (iii) Only one ligand-binding site is predicted per target sequence—the site with the largest identified ligand cluster. However, the server does also provide the data showing all putative ligand clusters, and these clusters can be made visible to users on the results page using the Jmol plugin. (iv) The FunFOLD2 server currently outputs predictions based on the best predicted IntFOLD2-TS model, and the top predicted model may not always be the best model.

## CONCLUSIONS

The FunFOLD2 server provides biologists with an intuitive interface for the prediction of protein–ligand interactions from amino acid sequences. Graphical output and plug-ins are provided to facilitate interactive visualization of predicted interactions in 3D, and machine-readable files are provided for developers. The algorithms within the FunFOLD2 server have been independently tested in the recent international CASP10 competition where the method was found to rank amongst the top few. Additionally, both the FunFOLD (2) and FunFOLDQA algorithms have been extensively benchmarked on both the CASP8 and CASP9 data sets (1,2).

## SUPPLEMENTARY DATA

Supplementary Data are available at NAR Online: Supplementary Methods.

## FUNDING

Studentship from the University of Reading, MRC Harwell and the Diamond Light Source. (to M.T.B.). This research leading to these results has received funding from the European Union Seventh Framework Programme [FP7/2007-2013] under grant agreement No. [246556 to D.B.R.]. Funding for open access charge: Genoscope, Institut de Génomique, Commissariat à l'Energie Atomique et aux Energies Alternatives. European Union Seventh Framework Programme [FP7/2007-2013] under grant agreement No. [246556].

*Conflict of interest statement*. None declared.

## References

[1] Roche DB, Buenavista MT, McGuffin LJ (2012). FunFOLDQA: a quality assessment tool for protein-ligand binding site residue predictions. PLoS One.

[2] Roche DB, Tetchner SJ, McGuffin LJ (2011). FunFOLD: an improved automated method for the prediction of ligand binding residues using 3D models of proteins. BMC Bioinformatics.

[3] Schwede T, Sali A, Honig B, Levitt M, Berman HM, Jones D, Brenner SE, Burley SK, Das R, Dokholyan NV (2009). Outcome of a workshop on applications of protein models in biomedical research. Structure.

[4] Buenavista MT, Roche DB, McGuffin LJ (2012). Improvement of 3D protein models using multiple templates guided by single-template model quality assessment. Bioinformatics.

[5] Matthews BW (1975). Comparison of the predicted and observed secondary structure of T4 phage lysozyme. Biochim. Biophys. Acta..

[6] Roche DB, Tetchner SJ, McGuffin LJ (2010). The binding site distance test score: a robust method for the assessment of predicted protein binding sites. Bioinformatics.

[7] Bindschedler LV, McGuffin LJ, Burgis TA, Spanu PD, Cramer R (2011). Proteogenomics and in silico structural and functional annotation of the barley powdery mildew Blumeria graminis f. sp. hordei. Methods.

[8] Fuller SJ, McGuffin LJ, Marshall AK, Giraldo A, Pikkarainen S, Clerk A, Sugden PH (2012). A novel non-canonical mechanism of regulation of MST3 (mammalian Sterile20-related kinase 3). Biochem. J..

[9] Gabanyi MJ, Adams PD, Arnold K, Bordoli L, Carter LG, Flippen-Andersen J, Gifford L, Haas J, Kouranov A, McLaughlin WA (2011). The structural biology knowledgebase: a portal to protein structures, sequences, functions, and methods. J. Struct. Funct. Genomics.

[10] Lopez G, Valencia A, Tress ML (2007). firestar–prediction of functionally important residues using structural templates and alignment reliability. Nucleic Acids Res..

[11] Brylinski M, Skolnick J (2008). A threading-based method (FINDSITE) for ligand-binding site prediction and functional annotation. Proc. Natl Acad. Sci. USA.

[12] Hernandez M, Ghersi D, Sanchez R (2009). SITEHOUND-web: a server for ligand binding site identification in protein structures. Nucleic Acids Res..

[13] Sankararaman S, Kolaczkowski B, Sjolander K (2009). INTREPID: a web server for prediction of functionally important residues by evolutionary analysis. Nucleic Acids Res..

[14] Wass MN, Kelley LA, Sternberg MJ (2010). 3DLigandSite: predicting ligand-binding sites using similar structures. Nucleic Acids Res..

[15] Lopez G, Maietta P, Rodriguez JM, Valencia A, Tress ML (2011). firestar–advances in the prediction of functionally important residues. Nucleic Acids Res..

[16] Roy A, Yang J, Zhang Y (2012). COFACTOR: an accurate comparative algorithm for structure-based protein function annotation. Nucleic Acids Res..

[17] Zhou H, Skolnick J (2013). FINDSITE(comb): a threading/structure-based, proteomic-scale virtual ligand screening approach. J. Chem. Inf. Model.

[18] McGuffin LJ, Roche DB (2010). Rapid model quality assessment for protein structure predictions using the comparison of multiple models without structural alignments. Bioinformatics.

[19] Zhang Y, Skolnick J (2005). TM-align: a protein structure alignment algorithm based on the TM-score. Nucleic Acids Res..

[20] Xu J, Zhang Y (2010). How significant is a protein structure similarity with TM-score = 0.5?. Bioinformatics.

[21] Schmidt T, Haas J, Gallo Cassarino T, Schwede T (2011). Assessment of ligand-binding residue predictions in CASP9. Proteins.

[22] Pedersen C, van Themaat EV, McGuffin LJ, Abbott JC, Burgis TA, Barton G, Bindschedler LV, Lu X, Maekawa T, Wessling R (2012). Structure and evolution of barley powdery mildew effector candidates. BMC Genomics.

